# Cognitive-motor multitasking in older adults: a randomized controlled study on the effects of individual differences on training success

**DOI:** 10.1186/s12877-022-03201-5

**Published:** 2022-07-15

**Authors:** Melanie Mack, Robert Stojan, Otmar Bock, Claudia Voelcker-Rehage

**Affiliations:** 1 Department of Neuromotor Behavior and Exercise, Institute of Sport and Exercise Sciences, University of Münster, Wilhelm-Schickard-Straße 8, 48149 Muenster, Germany; 2 Institute of Human Movement Science and Health, Chemnitz University of Technology, Thueringer Weg 11, 09126 Chemnitz, Germany; 3 Institute of Exercise Training and Sport Informatics, German Sport University, Am Sportpark Muengersdorf 6, 50927 Cologne, Germany

**Keywords:** Adaptive, Individualized, Dual-tasking, Aging, Ecological validity, Exercise, Physical activity, Cognition, Cognitive testing, Combined cognitive-motor intervention, Simultaneous

## Abstract

**Background:**

Multitasking is an essential part of our everyday life, but performance declines typically in older age. Many studies have investigated the beneficial effects of cognitive, motor and combined cognitive-motor training on multitasking performance in older adults. Previous work, however, has not regarded interindividual differences in cognitive functioning and motor fitness that may affect training benefits. The current study aims to identify whether different training programs may have differential effects on multitasking performance depending on the initial level of cognitive functioning and motor fitness.

**Methods:**

We conduct a 12-week single-blinded randomized controlled trial. A total of *N* = 150 healthy older adults are assigned to either a single cognitive, a single motor, or a simultaneous cognitive-motor training. Participants are trained twice per week for 45 min. A comprehensive test battery assesses cognitive functions, motor and cardiovascular fitness, and realistic multitasking during walking and driving in two virtual environments. We evaluate how multitasking performance is related not only to the training program, but also to participants’ initial levels of cognitive functioning and motor fitness.

**Discussion:**

We expect that multitasking performance in participants with lower initial competence in either one or both domains (cognitive functioning, motor fitness) benefits more from single-task training (cognitive training and/or motor training). In contrast, multitasking performance in participants with higher competence in both domains should benefit more from multitask training (simultaneous cognitive-motor training). The results may help to identify whether tailored training is favorable over standardized one-size-fits all training approaches to improve multitasking in older adults. In addition, our findings will advance the understanding of factors that influence training effects on multitasking.

**Trial registration:**

DRKS (German Clinical Trials Register), DRKS00022407. Registered 26/08/2020 - Retrospectively registered at https://www.drks.de/drks_web/setLocale_EN.do

## Background

Multitasking (MT) is an integral part of our daily life. Driving a car while talking with a passenger, or strolling on the sidewalk while watching shop windows, are often-cited examples of everyday behavior in which we execute multiple actions concurrently. A large number of studies evaluated how such concurrent cognitive-motor actions are scheduled, coordinated and supervised [1]. Initial theoretical concepts proposed an unitary, high-level cognitive mechanism called “supervisory attention system” [2] or “central executive” [3]. This mechanism has later been partitioned into distinct cognitive functions, including mental set shifting, information updating, and inhibition of prepotent responses, which are summarized under the umbrella term “executive functions” [4, 5], although the existence of distinct and separable executive functions has lately been called into question [6]. It is well established that MT skills deteriorate in older age (e.g., meta-analysis by [7]), particularly when tasks place a high demand on working memory [8] or on visuo-spatial processing [9]. This deterioration has been attributed to an interrelated decay of perceptual, sensorimotor and cognitive functions in older age [10], which have been shown to differ considerably between individuals [11]. We aim to evaluate training programs including cognitive exercises or motor exercises or both, which are already known to counteract the age-related decay of MT, taking into account interindividual variability.

When performing two tasks simultaneously, performance decrements occur in either one or both tasks. Those relative performance decrements under MT compared to single-task (ST) conditions are known as MT costs (MTC). A range of studies aiming to reduce MTC in healthy young and older participants demonstrated that extensive practice led to a substantial reduction of MTC. In some cases, MTC were eliminated completely (review in [1]), e.g. if perfect time-sharing of two tasks is theoretically possible as it could be the case for choice reaction time tasks [12]. Transfer of benefits to unpracticed tasks has also been observed, which suggests that training can optimize not only the constituent tasks, but also the executive processes that supervise MT [13–16].

Numerous experimental studies with older participants evaluated various types of cognitive-motor MT training such as aerobic and resistance exercises alternating with visual discrimination tasks [17], strength and balance exercises simultaneous with calculation, visual search, and verbal fluency tasks [18], or seated stepping exercises simultaneous with verbal fluency tasks [19], and its effects on unpracticed MT scenarios. Here we focus on studies which included specifically the transfer of training to unpracticed cognitive-motor MTs, such as walking on a treadmill while completing a working memory test. Several systematic reviews [20–26] summarized the literature in this area, each from a somewhat different viewpoint such as fall prevention [26], motor and cognitive functions [24], or physical functioning [20]. When the literature covered by those reviews is adjusted by removing (1) double citations of the same study, (2) studies that didn’t test for transfer to unpracticed cognitive-motor MTs, (3) studies that didn’t include an adequate control group, and (4) studies of populations with health problems (frail, balance-impaired, osteoarthritis, osteoporosis), then 25 studies remain. Adding four more recent studies [17, 27–29] brings the total up to 29.

All 29 intervention studies identified from those reviews, included cognitive-motor MT in their pre- and posttests, but only 15 of them also included cognitive-motor MT as a substantial or as the only content of their training regime; we will refer to them as MT training studies. The other 14 studies trained mainly or exclusively ST (two studies ST cognitive training and twelve studies ST motor training); we will refer to them as ST training studies. Eleven of the 15 MT training studies and nine of the 14 ST training studies found a statistically significant reduction of MTC. In particular, MTC for the motor component decreased after training in seven MT training studies and in six ST training studies; MTC for the cognitive component decreased after training in six MT training studies, and in three ST motor training studies (cognitive MTC were not provided in four MT training and in five ST training studies). Ten studies compared MT training and ST training [18, 19, 27–34], of which seven of them found MT training more beneficial as ST training [18, 19, 27, 29, 31, 33, 34]. Only two studies compared MT training with fixed task priority and MT training with variable task priority; they found the latter instruction to be more effective [15, 35].

The heterogenous results on training success in above studies led to a widespread discussion about possible causes and remedies. In particular, it was proposed that some studies yielded no training benefits because of shortcomings regarding (1) sample size: some studies trained only a small number of participants such that, even if training benefits existed, they were not likely to reach statistical significance; (2) training quantity: in some studies, the duration, number and/or frequency of training sessions was quite low, and those studies therefore possibly suffered from floor effects, i.e., the intensity threshold for substantial training benefits was not reached; (3) training intensity: some studies implemented less-demanding tasks, and therefore again potentially suffered from floor effects; (4) training variety: some studies trained only a single combination of tasks, which might not be the best way to support transfer [20–26].

The present work addresses yet another possible shortcoming: participants’ initial competence. Since interindividual differences in cognitive functioning as well as in motor fitness increase with advancing age [36], it is quite likely that some participants in the above studies had higher cognitive functioning and/or motor fitness, while others had lower cognitive functioning and/or motor fitness. It further is conceivable that individuals’ initial cognitive and motor competence affect training gains [37–40]. Older adults with lower cognitive and motor competences may benefit less from training as a result of a need to master a certain skill level in order to achieve training gains. On the other hand, lower cognitive and motor competences could also lead to higher gains, as these individuals offer more need for improvement. In addition, training gains might be further moderated by additional variables, such as education, gender, or age, which we will not specifically address in our study. If so, the limited success in above studies could simply reflect a sampling bias: possibly, some studies happened to recruit a larger portion of participants with lower cognitive functioning and/or motor fitness, which could explain why they didn’t yield significant training benefits.

If participants’ initial competence indeed plays a role, this should be considered when designing new training regimes. In particular, persons with lower cognitive functioning should first be given cognitive training alone, before simultaneous cognitive-motor training is introduced. Similarly, persons with lower motor fitness should start out with motor training alone, and those with lower cognitive functioning and motor fitness should start out with a combination of cognitive training alone and motor training alone. Only persons with higher cognitive functioning and motor fitness should receive simultaneous cognitive-motor training right away. Such an approach would be in line with the modern concepts of “individualized”, “personalized”, or “tailored” training. According to those concepts, physical [41, 42], cognitive [43, 44], psychosocial [45], and other forms of interventions should not use off-the-rack standardized protocols, but rather should be fitted to each participant’s initial level of competence. The purpose of our study is to provide experimental evidence for or against the potential benefits of tailored training on cognitive-motor MT.

Available literature provides some indirect evidence in favor of tailored training. For example, older adults’ reduced motor fitness was found to be associated with reduced automation and increased cognitive control in gait tasks [46]. This fits well with the view that in older age, more cognitive resources must be allocated to the motor system and thus are no longer available for the supervision of MT [47]. It has further been shown that motor training of older adults reduces their need for cognitive control of gait [48]. This could indicate that motor training frees up some of the cognitive resources which older persons otherwise would allocate to motor control, such that the freed-up resources then become available for the supervision of MT.

To acknowledge the interindividual differences and evaluate the role of initial cognitive and motor competence, we designed an experimental protocol which scrutinizes whether older persons with lower initial competence in either the cognitive and/or the motor domain benefit more from domain-specific ST training, while those with higher competence in both domains benefit more from MT training. If our data meet these expectations, this would strongly support the use of tailored training regimes for cognitive-motor MT in older age. The training regimes in our study follow established cognitive and/or motor training procedures. Training benefits on participants’ MT performance are assessed by a MT walking test and by a MT driving test in a virtual environment. The latter test was designed to mimic the cognitively demanding behavior in everyday life.

## Methods

### Study aims

This study aims to investigate whether three different training programs (cognitive, motor, and simultaneous cognitive-motor training) have differential effects on MT performance, in dependence on participants’ initial cognitive functioning and motor fitness. Specifically, we expect that (H1) for participants with lower cognitive functioning, MT performance benefits more from cognitive training than from motor or simultaneous cognitive-motor training, (H2) for participants with lower motor fitness, MT performance benefits more from motor training than from cognitive or simultaneous cognitive-motor training, and (H3) for participants with higher motor fitness and higher cognitive functioning, MT performance benefits more from simultaneous cognitive-motor training than from motor or cognitive training.

### Study design

A single-blind, randomized, controlled intervention with healthy older adults is conducted. Three training programs (cognitive, motor, and simultaneous cognitive-motor training) are included, as well as pre- and posttests. All participants complete the same pre- and posttests. An overview of the study design is presented in Table 1. This study protocol follows the SPIRIT guidelines [49, 50]. The trial is retrospectively registered in the DRKS (German Clinical Trials Register) at 26/082020 with the registration number DRKS00022407.

**Table 1:**
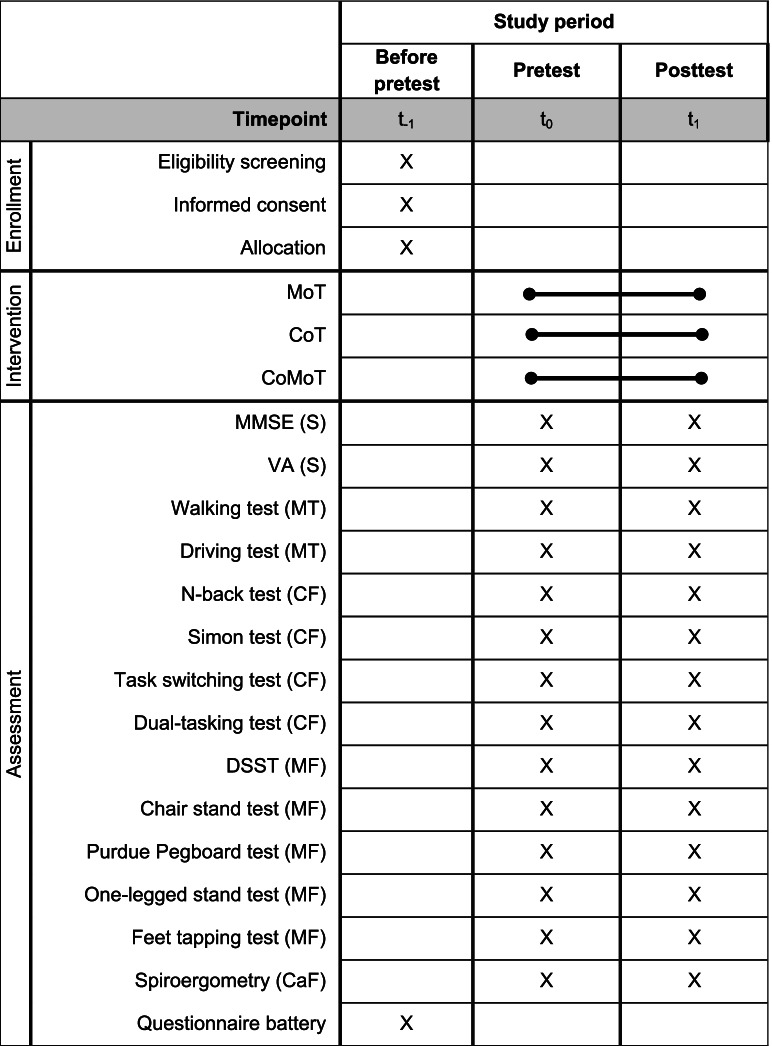
Spirit diagram: Schedule of activities

*X *implementation of the assessment, the line with two dots represents period, starting point and endpoint of the trainings, *t*_-1 _before pretest, *t*_*0 *_at pretest, *t*_*1 *_at posttest, *MoT *Motor training, *CoT *Cognitive training, *CoMoT *Simultaneous cognitive-motor training, *S *Screenings, *MT *Multitasking, *CF *Cognitive functions, *MF *Motor fitness, *CaF *Cardiovascular fitness, *MMSE *Mini-Mental State Examination, *VA *Visual acuity test, *DSST *Digit Symbol Substitution Test

### Recruitment of participants

This study is conducted at the University of Münster (WWU), and at the Chemnitz University of Technology (TUC), Germany. Participants are recruited via homepage announcements, by personal contact, senior college, local sports clubs as well as by advertisements in local newspapers, radio stations, and flyers. Interested participants are screened for eligibility using a standardized telephone interview that surveys the predefined inclusion and exclusion criteria as described below. All participants give written consent prior to study enrolment. The consent from will be sent home to them together with other study material. All participants receive monetary compensation (15 € per testing day).

### Eligibility criteria

Inclusion criteria are: (1) aged between 65 and 75 years (minor exceptions are made for couples for ethical reasons: < 65 and > 75 years), (2) right-handed, (3) active car driving (e.g., at least once a week within the last 6 months), (4) ability to walk unassisted without self-reported problems (e.g., difficulty to breath, pain, and cardiac palpitations), and (5) community-dwelling. Exclusion criteria are: (6) BMI > 30, (7) red-green deficiency or red-green-color blindness, (8) orthopedic impairments, (9) perceived health concerns, (10) neurological diseases, (11) cardiovascular disorders, (12) previous heart attack or stroke, or (13) previous head/brain surgery. All information on inclusion and exclusion criteria is self-reported during a telephone interview.

### Randomization

Each subject is randomly allocated to a training group by assigning a random number between 1 and 3 (1 = motor training, 2 = cognitive training, 3 = simultaneous cognitive-motor training) to each participant using Microsoft Excel. Couples are allocated to the same training program to ensure that they can train together by assigning only one number. Participants are informed about their training program after pretesting.

### Anonymization and blinding

Different blinding procedures are applied to avoid performance bias during data collection and training, and confirmation bias in data analysis and data collection. Staff for data collection and data analysis is blinded for participants’ training group. Trainers are blinded for participants’ pretest performance and are not involved in posttesting.

Anonymity is ensured by utilizing only pseudonymized codes (IDs) to document pre- and posttest performance. For training documentation, only participants’ names are used. For the cognitive training and the simultaneous cognitive-motor training, trainers additionally possess participant’s login data of the cognitive training software. For data analysis, names and IDs are assigned by a key-list to which only the current study coordinators and the principal investigators have access.

### Instruments and measurements

#### Screenings

*Cognitive impairment* is screened using the Mini-Mental State Examination (MMSE, [51]). The test covers different cognitive domains, such as attention, arithmetical skills, registration, language, memory, and orientation. Cognitive impairment is measured on a 30-point scale (30 = no cognitive impairment) with a score < 25 indicating mild cognitive impairment [52].

*Visual acuity* is screened using the Freiburg Visual Acuity Test (FrACT v 3.9.3, [53]; https://michaelbach.de/fract/) with a cutoff score of 20/60. Participants are seated on a chair that is positioned in 3 m distance from the computer monitor. Eighteen Landolt rings (circles with a small opening in one out of eight possible directions) are displayed sequentially on a 24″ monitor (1920 × 1080). Each Landolt ring opening is paired with a number from 1 to 8 that are displayed on a DIN A4 paper sheet above the monitor. Participants are asked to state the number that matches the opening of the Landolt ring. The size of the circles changes with response success. Both decimal acuity (VAdec) and logarithm of the minimum angle of resolution (LogMAR) are calculated.

Participants with cognitive impairment (MMSE < 25) and a visual acuity below 20/60 are excluded from further data analysis.

#### Sociodemographic and psychological questionnaires

All participants complete a self-administered questionnaire battery, which assesses the following outcome parameters: personal and sociodemographic information (age, gender, weight, height, etc.), recent driving behavior, years of education and employment, subjective health, objective health, smoking behavior, history of falls, fall efficacy, physical activity, social and leisure time activities, use of electronic devices and computer, subjective hand use, handedness, and personality. The questionnaire battery comprises validated instruments (partly modified), which are shown in Table 2, and some self-generated items. Participants are asked to complete the questionnaire battery at home, and to hand them over to the experimenter on their first day of testing.

**Table 2:** Instruments used for the questionnaire battery

Outcome measure	Instrument
Subjective health	Self-rated health [54]
Objective health	Diseases and use of medication [55]
Smoking behavior	Tobacco consumption [56]
History of falls	Elderly Fall Screening Test [57]
Falls efficacy	Falls Efficacy Scale [58]
Physical activity	Baecke Inventory [59]
Social and leisure time activities	Participation in everyday activities [60]
Subjective hand use	Frequency of hand use in different daily activities [61]
Handedness	Edinburgh Handedness Inventory [62]
Personality	Big Five Inventory [63]

#### Multitasking test: virtual reality walking

##### Hard- and software

This test is performed with the GRAIL system (Gait Real-time Analysis Interactive Lab, Motekforce Link, Amsterdam, the Netherlands), which is a valid and reliable device to assess gait [64]. It comprises a 3D instrumented split-belt treadmill (0.8 × 1.5 m) with two embedded force plates, a semi cylindrical 240° projection screen (2.4 × 5 m), and a Vicon MX optical infrared tracking system (Vicon, Oxford, United Kingdom). The participant is secured by two handrails laterally attached to the treadmill, two laser barriers at the front and back end of the treadmill, and a safety harness that is attached to the ceiling to prevent participants from falling. In addition, the experimenter can press a stop button to instantly stop the treadmill in case of emergency. Four serially-connected RGB projectors project a virtual scenario on the projection screen. A photodiode is attached to the screen to accurately measure stimulus onsets and to prevent varying projection onsets. An ergonomic handheld key switch with a left and a right button and a voice recorder are used to record participant’s responses.

**Fig. 1:**
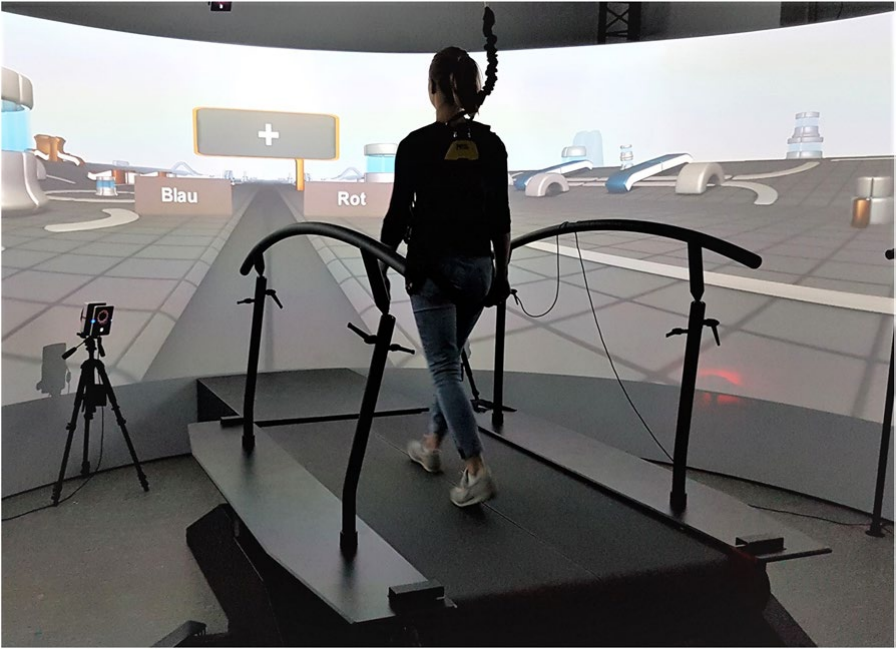
Gait Real Time Analysis Interactive Lab (GRAIL), customized MT scenario

The virtual scenario is designed with D-Flow (Motekforce Link, Amsterdam, the Netherlands). The scenario roughly depicts an industrial-like environment with abstract objects placed laterally to a virtual walking path (see Fig. 1). Motor and cognitive tasks are customized and added to the application (cf. below). All instructions and all stimuli are presented visually at eye level in a small, rectangular area in the middle of the projection screen.

##### Motor and cognitive tasks

Six different tasks with five trials each are presented in a mixed order. Task presentation and order is identical for each participant and at pre- and posttest. No given task is presented more than twice in a row. Every trial lasts 30 s with inter-trial intervals of 3 s to introduce the next trial (e.g., “Standing only” or “Walking only”, in German language). The scenario lasts 16.5 min in total.

One baseline task, three STs (one motor, two cognitive), and two cognitive-motor MTs are performed: (1) Standing task (baseline): Participants stand quietly with both feet on the treadmill while maintaining a straight direction of view by looking at a fixation cross. Posture is assessed via ground reaction forces. (2) Walking task: Participants walk at 1 m/s while maintaining a straight direction of view by looking at a fixation cross. As the treadmill accelerates and decelerates at 0.2 m/s^2^, transitions between standing and walking take 5 s. Gait performance is assessed via ground reaction forces. (3) Serial Threes task: The Serial Threes task is an established measure of updating of working memory [65, 66]. Participants stand quietly on the treadmill and look at a fixation cross at the center of the projection screen. At the beginning of each trial, a three digit-number is displayed for 5 s. Participants are asked to count backwards in steps of three loudly, and as quickly and accurately as possible. They are instructed to keep their eyes open during counting, and to stop counting when the next task is displayed on the screen. Participants are asked to spell out the whole number (e.g., “177” instead of “77”), and not to correct errors (i.e., to continue counting backwards from a possibly wrong number). Verbal responses (i.e., numbers) are protocolled by the experimenter, and are additionally recorded using a voice recorder. (4) Color Word Stroop task: The Stroop task is used to assess inhibitory control [67]. In each trial, color-denoting words (i.e., yellow, red, blue, green) are sequentially presented for 500 ms in a mixed order with ten words per trial. Each stimulus is followed by a central fixation cross for 1800 to 2200 ms, such that mean ISI is 2500 ms. Font and meaning of color words match on congruent trials (e.g., the word “green” in green font), and do not match on incongruent trials (e.g., the word “green” in blue font). Two response words are presented for 1500 ms on the projection screen, time locked with stimulus onset. They are displayed in two rectangular areas, one located to the left below the stimulus word and the other located to the right below the stimulus word. Both response words are presented in white font; one names the font of the stimulus word, and the other names one of the three other fonts. Participants have to indicate which of the two response words names the stimulus font, by depressing either the left or the right button of a handheld key switch. Participants are instructed to give their responses as fast and as accurately as possible. The design of the Stroop task is balanced across congruency (50% congruent, 50% incongruent), stimulus font (25% of each font), stimulus meaning (25% of each meaning), position of correct and false answer boxes (50% left, 50% right), and frequency of correct and false response words per color (50% correct, 50% false). Reaction times and correctness of responses are recorded. (5) Multitask 1: The walking task and Serial Threes task are executed concurrently. (6) Multitask 2: The walking task and Stroop task are executed concurrently. Participants are instructed to not give preference to either one of the concurrently executed tasks, while responding to cognitive tasks as fast and as accurately as possible. Outcome measures are the same as described above.

##### Procedure

Participants familiarize with the treadmill by walking through a virtual forest environment for about 5 to 10 min while walking speed increases slowly up to 1 m/s. Familiarization ends when participants are able to walk securely while focusing their attention on the center of the projection screen. Physically low demanding tests (including cognitive functions test such as MMSE and DSST), of about 12 to 15 min duration in total, are scheduled after familiarization to ensure that participants return to a physical resting state. After that, participants perform a short practice run of about 2 min including a shortened trial of each task in a fixed order.

#### Multitasking test: virtual reality driving

##### Hard- and software

This test follows closely the driving test of Wechsler et al. [68], where a schematic drawing of the setup is provided. The setup consists of a VW Golf seat and three 48″ monitors that are mounted at eye level, covering a visual field of 195°. A Logitech G27 steering wheel is located slightly to the left in front of the middle monitor. Gas and brake pedals are placed on the floor in a position similar to a real car. The car seat and pedals are adjustable to provide a realistic and comfortable driving position. A conventional numeric keypad is mounted to the right of the steering wheel, within participants’ easy reach. Numbers from 1 to 6 are visible on the keypad, other characters are covered by black tape. A regular headset is used for auditory task presentation and to present characteristic driving noise.

**Fig. 2:**
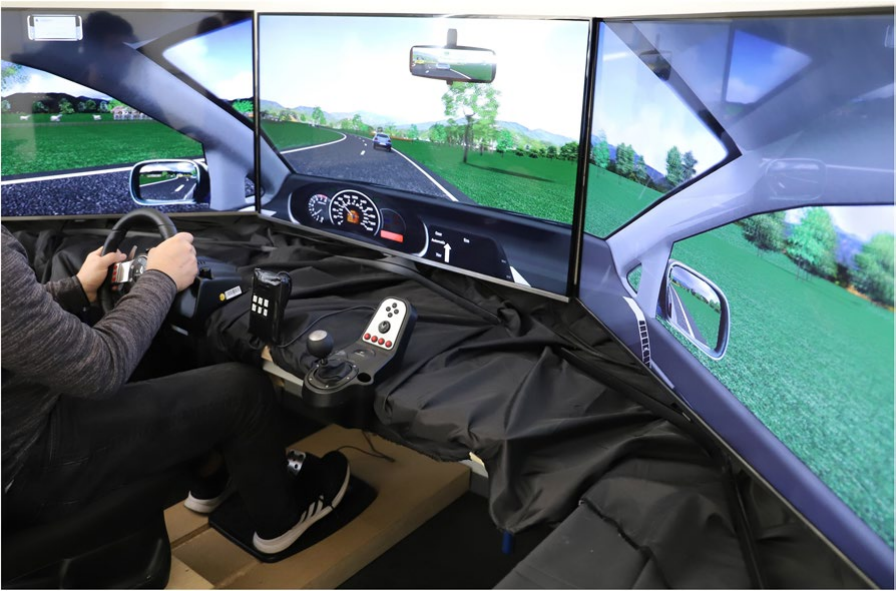
Carnet Soft Driving Simulator, customized MT scenario

The driving simulation uses commercially available hard- and software (Carnetsoft version 8.0, Groningen, The Netherlands). Figure 2 shows the setup of the scenario. It displays 25.7 km of a slightly winding rural road, without intersections or traffic lights. The simulated environment pictures a typical landscape with clouds in a blue sky, mountains, little animal enclosures, grasslands, trees, traffic signs, gas stations, and construction sides. Regular oncoming traffic comprises cars and busses. The scenario does not involve any pedestrians, cyclists, or other road users. Participants drive a VW Golf with automatic transmission and a simulated dashboard that is presented at the bottom of the middle screen. Velocity is displayed on a speedometer. The participant’s vehicle is continually accompanied by one car in the rear and another car in front. The lead car is programmed to drive at 70 km/h, and to slow down slightly if the distance to the participant’s car exceeds 100 m. The rear car is programmed to follow at a reasonable distance. In case of an accident, the front window shatters and the driver’s car is directly thereafter repositioned between the lead car and the rear car.

##### Motor and cognitive tasks

Participants perform a driving task and a battery of additional tasks that are designed to mimic cognitively effortful activities typically performed during driving. On three driving courses, they perform the driving task alone, the additional tasks alone, or the driving and the additional tasks concurrently.

Driving-alone course: participants are instructed to follow the lead car at a regular distance with about 70 km/h, to pay attention to posted speed limits, and to brake when the lead car brakes. Ten braking events are presented at irregular course locations, that are identical for all participants and at pre- and posttest. As the lead car approaches a 40 km/h speed limit sign, its brake lights flash up and the car slows down to 40 km/h within about 7 s. It keeps that velocity for about 6 s, and then accelerates within about 9 s back to 70 km/h. Lateral car position, car velocity, and distance to the lead car are continuously assessed. For braking responses, reaction times for gas-off and brake-on reactions are measured.

Cognition-alone course: the participants’ car drives in autopilot mode and responds automatically to braking maneuvers of the lead car. Two different types of tasks are presented at fixed course locations in a mixed order that is identical for all participants and at pre- and posttest. Both tasks are presented either visually on the windshield or auditory through headphones. (1) In the typing task, participants are asked to type a three-digit number with their right hand into the numeric keypad, as quickly and as accurately as possible. Stimuli presentation lasts about 5 s for visual trials, and about 3 s for auditory trials. (2) In the reasoning task, participants are asked to verbally provide arguments for or against issues of general interest, e.g., to state an argument for/against the use of electric vehicles. Each request is presented for about 5 s visually or 4 s auditory, and cannot be answered adequately by a simple “yes” or “no”. Requests are limited to 80 characters, and to two lines on the windshield. The experimenter protocols the participants’ answers. Answers are marked as correct if participants give a valid argument, and are marked as incorrect if participants give an invalid argument, or if they do not answer at all. The validity of arguments is agreed-upon among experimenters before the study. Both the typing and the reasoning task comprise 30 trials, 15 presented auditory and 15 presented visually. The concrete stimuli (three-digit numbers, reasoning questions) differ between trials, and they also differ between pre- and posttest; however, they are the same for all participants. Reaction times and correctness of responses are measured for the typing task, and correctness of answered requests is assessed for the reasoning task (as reaction time was not assessed).

MT course: participants actively drive and brake for the lead car, and they also respond to typing and reasoning tasks that are analogous to those on the cognition-alone course. No instructions are given regarding the preference for driving versus for additional tasks.

##### Procedure

One half of the driving-alone course and the complete cognition-alone course are scheduled on 1 day, in balanced order. The other half of the driving-alone course and the complete MT course are scheduled on another day, again in balanced order. The order of days is also balanced. On a separate day before testing, participants practice the driving-alone course and the cognition-alone course for approximately 5 min each, but they don’t practice the MT course.

#### Tests of cognitive functions

A battery of five tests is used to measure a broad range of different cognitive functions. All tests follow standardized procedures and instructions. Four tests are computerized, one is a paper and pencil test. Computerized tests are conducted on a 24″ computer screen with a display resolution of 1920 × 1080 pixel and a screen distance of about 65 cm. Each computerized test takes about 10 min with up to three practice trials of about 1 to 2 min each. Response feedback in provided after practice trials, but not after registered trials.

The N-back, Simon and Task switching tests are programmed in E-Prime 2.0 (Psychology Software Tools, Pittsburgh, PA) with stimuli presented in six blocks with inter-block breaks of 5 s (20 s after block 3). The maximum response window is 2000 ms. After a response is given or after 2000 ms a central fixation cross is presented for a variable response-stimulus interval between 800 to 1200 ms. All stimuli are black and presented on a white screen background. Participants respond by depressing the “X” or “M” key on a German keyboard with their left and right index finger, and they are instructed to respond as fast and as accurately as possible. Reaction times and correctness of responses are recorded.

*Working memory updating* (‘updating’) is assessed using the 2-back condition of a visuo-spatial N-back test [69]. A black 4 × 4 grid is presented continuously. Dots (*n* = 19 per block) are presented sequentially in the center of different grid cells for 500 ms. Participants have to memorize the position of the dots and to depress the right key “M” when the position of the current dot is identical to the position of the second-to-last dot (target). They have to depress the left key “X” when the current dot appears at a different position as the second-to-last dot (non-target). In total, 30 targets and 72 non-targets are presented.

*Inhibitory control* is assessed using the Simon test [70]. A black fixation cross is presented continuously on a white screen. Left- or rightward pointing arrows (*n* = 32 per block) are displayed sequentially for 500 ms either on the left or right side of the fixation cross. For one half of the stimuli the direction and position of the arrow are congruent (e.g., leftward pointing arrow on the left side), while for the other half of the stimuli, direction and position are incongruent (e.g., leftward pointing arrow on the right side, *n* = 96). Participants are instructed to press the left key “X” for leftward pointing arrows and the right key “M” for rightward pointing arrows.

*Shifting* is assessed using a modified visual task switching test [71]. Geometrical shapes (*n* = 17 per block) are presented sequentially for 1500 ms. The geometrical shapes are either quadratic or circular and either big or small. Participants are instructed to indicate either the size of the shape (subtask A) or the form of the shape (subtask B) by pressing either the left key “X” for small or circular shapes or the right key “M” for big or quadratic shapes. In each block subtasks are presented in the following order: AABBAABBAABBAABBA. No external cues about subtask order are provided.

*Dual-tasking* (DT) is assessed using a dual-tasking test adapted from literature [72] where a manual tracking task and an auditory discrimination task are performed concurrently. Nine trials with about 45 s each are presented in three blocks: (a) three trials ST manual tracking (b) three trials ST auditory discrimination, and (c) three trials DT manual tracking and auditory discrimination with both tasks being performed simultaneously. The three blocks are presented in a randomized order across participants. In ST manual tracking trials, a small red target square moves from one side of the screen to the other following an unpredictable wave-shaped path. Participants are instructed to track the red target with a small white crosshair cursor that is controlled using a joystick. Only the vertical movement of the cursor can be controlled, the horizontal movement is aligned with the target. Participants are instructed to use the joystick with the right hand to keep the cursor as close as possible to the target. The vertical distance between cursor and target is continuously measured over the whole trial. In ST auditory discrimination trials, ten target sounds and 18 to 20 distractor sounds are presented per trial in a random sequence through headphones: the target sound is a high-pitched tone (1086 Hz), and two low-pitched tones (217 Hz and 652 Hz) are non-target distractor sounds. All sounds are presented for 75 ms with a jittered ISI of 1000 to 1300 ms. Participants are instructed to respond to the high-pitched tone only by depressing the “F12” key with their left index finger, and to react as fast and as accurately as possible. Reaction times and correctness of responses are assessed. In the DT manual tracking and auditory discrimination trials, participants perform both tasks simultaneously. No instructions are given regarding the prioritization of either one of the tasks. Same outcome measures are recorded as in the ST conditions.

*Global cognition* is assessed using the Digit-Symbol-Substitution test (DSST [73]). This test is part of the Wechsler Adult Intelligence Scale [74] and is performed as paper-and-pencil tests. It consists of nine digit-symbol pairs followed by a list of digits with blank cells below. Participants are required to write the corresponding symbol in each blank cell below the digits as quickly as possible. The number of correct symbols within 90 sec is analyzed.

#### Tests of motor fitness

A battery of four established motor tests is used to determine different aspects of motor fitness [60, 75] following standardized procedures and instructions. Time is kept using a regular stop watch. Short practice trials with two to five repetitions are performed before each test.

*Leg strength and endurance* is assessed using the Chair stand test of the senior fitness test for older adults [76]. Participants sit on a height-adjustable chair without armrests. Arms are crossed with hands on opposite shoulders. Participants continuously rise up to a straight standing position and sit down to a fully seated position with a straight back as often as possible within 30 s. They are asked to keep their arms crossed and both feet on the floor during the whole test. Correctly executed chair stands are registered.

*Bimanual dexterity* is measured with the Purdue Pegboard test [77, 78]. The Pegboard consists of two rows of 25 small holes from top to bottom. Small metal pins (pegs) are located at the upper left and right of the board. Participants are instructed to simultaneously pick up a peg from the right side with the right hand and a peg from the left side with the left hand, to place both pegs into the top empty holes in the left and right row, and to repeat this procedure as often as possible within 30 s. Three trials are performed. The number of rows with two correctly placed pegs is assessed for each trial.

*Static balance* is assessed using the One-legged stand test with open and closed eyes [79]. The test is performed in the GRAIL (cf. section on virtual reality walking test), but the waistcoat is not used as it could affect participants’ posture. Eight trials are performed in total, the first four trials with eyes open, and the second four trials with eyes closed. Each leg is assessed twice, in alternating order. Participants stand on one leg with the other leg slightly flexed while looking straight ahead. The experimenter stands quietly sideways to the participant, to prevent falls. Participants are instructed to keep their arms at the side of their body, to not hop with their standing leg, not to put down their lifted feet, not to push the lifted leg against the standing leg during balancing, and not to open their eyes during eyes closed trials. Each trial is self-initiated. Participants are instructed to stand on one leg as long as possible. The experimenter starts time keeping when the participant lifts one leg, and stops when the participant is violating one of the above mentioned standards or after 20 s. Standing duration and ground reaction forces are assessed.

*Psychomotor speed* is measured using the Feet tapping test [75]. Participants sit on a stationary chair (height adjustable) without armrests, and are asked to move both feet simultaneously back and forth across a mid-sagittal line on the floor. They are instructed to move both feet as fast as possible, while ensuring that both soles completely contact the floor at each tap. Two trials of 20 s duration are performed. The number of correct taps is registered using a hand clicker.

#### Assessment of cardiovascular fitness

Cardiovascular fitness is measured by a spiroergometry (ZAN600 CPET, nSpire Health, Oberthulba, Germany) on a stationary bicycle (Lode Corival cpet, Groningen, the Netherlands). Participants are asked to avoid caffeine and alcohol intake for 12 hrs before testing and any vigorous exercise on the day before. Each measurement is either accompanied by a physician or participants are required to bring a medical clearance certificate based on exercise electrocardiography (ECG) and clinical history. Respiration (oxygen (VO_2_) and carbon dioxide consumption (VCO_2_)) is measured breath-by-breath. Heart rate is assessed using an integrated digital twelve-lead electrocardiogram (Kiss, GE Healthcare, Munich, Germany). A Borg’s 6–20 scale [80] is used to assess the rate of perceived exertion (RPE, 6 = “No exertion at all”, 20 = “Maximal exertion”) every 2 min [80] as indicated by the participant by pointing on the number from 6 to 20 on an RPE sheet. Blood pressure is monitored via a sphygmomanometer. Participants undergo a ramp protocol. For male participants, the load starts at 20 W and continuously increases by 20 W/min. For female participants, the load starts at 10 W and increases by 15 W/min. All participants are instructed to maintain a cycling frequency between 60 to 80 rpm. Both protocols are preceded by a 3 min resting period and followed by 5 min cool-down period (1 min initial load, 4 min no load). Protocols are terminated when participants respiratory exchange ratio (RER = VCO_2_/VO_2_) remains > 1.05 for at least 30 s or exceeds 1.10, in case of volitional fatigue, or occurrence of physiological risk factors (i.e., blood pressure > 230/115 mmHg, dizziness, HR > about 220-age, cardiac arrhythmia, or other abnormalities). Each measurement is accompanied by an experienced sport scientist. Peak oxygen uptake (VO_2_ peak: VO_2_ consumption during the maximum load level achieved), RER, and the maximum load level (i.e., wattage) are analyzed and considered for rating the measurement validity.

### Training

Three different training programs (cognitive, motor, and simultaneous cognitive-motor training) are conducted over a period of 12 weeks in the facilities of the TUC and the WWU. Two training sessions are scheduled per week, for a total of 24 training sessions (total training time: 1080 min). Each training session has a duration of about 60 min, including 15 min for preparation (e.g., changing clothes, warm-up, hard- and software preparation). Each training program is conducted as circuit training with three 15 min blocks yielding a training sequence with a total of 72 training blocks. To ensure continuous training progress, difficulty level of the training is continuously adapted to the individual’s performances. The training is not intended to improve participants’ cardiovascular fitness, especially to ensure that the hypothesized effects of the applied training programs do not interfere with the effects of cardiovascular training on cognitive and brain function. Therefore, participants of the motor and the simultaneous cognitive-motor training wear a heart rate monitor during training to ensure that training intensity does not exceed 60% of VO2-peak, as determined by spiroergometry at pretesting. Training sessions are supervised by skilled trainers in group settings with a trainer-participant-ratio of at least 1:3 (motor and simultaneous cognitive-motor training) or at least 1:10 (cognitive training). The trainers provide instructions, help participants to sign into the software applications, answer questions and protocol participants’ performances. They are onboarded to the training procedure in a two-day workshop. To improve attendance and contribution, explanations about the possible benefits of the training are provided to the participants. Attendance and drop-outs are documented. To ensure a total attendance of 24 sessions for each participant, missing training sessions (e.g., in case of illness) are made up within the total training period of 12 weeks. Apart from training, participants are asked not to change their regular everyday activities, including social, physical and cognitive activities. Table 3 illustrates exemplary training sessions for each of the three programs.

**Table 3:** Exemplary training session of the cognitive, motor, and simultaneous cognitive-motor training

Time (min)	Cognitive training	Motor training	Simultaneous cognitive-motor training
0–15	Preparation	Preparation	Preparation
15–30	**NeuroNation** Colorado (inhibitory control; dl 3)	**Treadmill training** Walking at an individually chosen pace (dl 1) Stop and start walking again (dl 2) Short and long steps alternating (dl 3)	**NeuroNation + treadmill training** Colorado (dl 1) + walking at an individually chosen pace (dl 1) Colorado (dl 1) + stop and start walking again (dl 2) Colorado (dl 1) + short and long steps alternating (dl 3)
30–45	**NeuroNation** Drehfluss (updating; dl 2)	**Floor training** Get up from a chair (strength; dl 2, balance board) Hip circles (flexibility) Semi tandem stand (balance; dl 1, AIREX pad) Knee lifts and external hip rotation (flexibility) Calf lifts (strength; dl 1, AIREX pad) Leg swinging sideward (flexibility)	**NeuroNation + floor training** Drehfluss (dl 2) + get up from a chair (dl 0, floor) Drehfluss (dl 2) + hip circles Drehfluss (dl 2) + semi tandem stand (dl 1, AIREX pad) Drehfluss (dl 2) + knee lifts and external hip rotation Drehfluss (dl 2) + calf lifts (dl 1, AIREX pad) Drehfluss (dl 2) + leg swinging sideward
45–60	**NeuroNation** Doppelmerker (updating, inhibitory control; dl 4)	**Treadmill training** Narrow and wide steps alternating (dl 4) Changing speed every 30 s (dl 5) Walking and lifting one knee sideways every 5th step (dl 6)	**NeuroNation + treadmill training** Doppelmerker (dl 1) + narrow and wide steps alternating (dl 4) Doppelmerker, (dl 1) + changing speed every 30 s, (dl 5) Doppelmerker, (dl 1) + walking and lifting one knee sideways every 5th step (dl 6)

NeuroNation: name of the software application; Colorado, Drehfluss, Doppelmerker: exercises within NeuroNation; *dl *difficulty level

#### Cognitive training

##### Training equipment and exercises

The training program is conducted in a computer-pool with one separate computer per participant. The exercises are presented on a computer monitor mounted at eye level in front of the participant. Handheld trackball mice (YUMQUA Y-01, YUMQUA, Shenzhen, China) were used to control the cursor. This ensures that the same pointing device can also be used for simultaneous cognitive-motor training (see below). The training program includes 22 different cognitive exercises from three different software applications NeuroNation (NeuroNation, Berlin, Germany; 15 exercises), Happyneuron (Scientific Brain Training, Lyon, France; four exercises), and Neuropeak ([81]; three exercises). Exercises train different cognitive functions, specifically inhibitory control, updating, shifting, MT and action planning which are essential for everyday life functioning. Each of these cognitive functions is trained to the same extend over the whole training course.

##### Procedure

Exercises are practiced and explained within the software. In each of the 72 training blocks one exercise is provided respectively. Over the whole training, the different exercises are performed several times in a predefined order which is similar for all participants. The first time an exercise is provided, it is performed at the lowest difficulty level and adapts automatically over the course of the training based on previous scores.

#### Motor training

##### Training equipment and exercises

The training program is conducted in a customized exercise room. Hand movements were not involved in the exercises, to ensure that the same exercises can also be performed during simultaneous cognitive-motor training (see below). Motor training consists of a floor program and a walking program. The floor program entails different exercises that train either strength (*n* = 10) or balance (*n* = 17). To vary the difficulty level of the exercises, different sport material is used: AIREX pad, balance board, balance pad with little nubs (Sissel Balance Fit), balance pad with big nubs (Sport-Thieme Gymfit, Germany), rocker board (easy version, Sport-Thieme Gymfit, Germany), rocker board (difficult version, Sport-Thieme Gymfit, Germany). In total, 29 variations of strength exercises and 51 variations of balance exercises are provided. Five different flexibility exercises are performed for recovery between and at the end of the strength and balance exercises: strength exercise (4 min), flexibility exercise (1 min), balance exercise (4 min), flexibility exercise (1 min), strength exercise (4 min), flexibility exercise (1 min). The walking program is conducted on a curved-belt non-motorized treadmill (Speedfit SpT-1000C, Tobeone, Korea). It comprises nine different walking exercises with varying difficulty. Exercises are changed every 5 min. The extent to which each of the three motor functions (walking = 435 min strength = 344 min, balance = 172 min is trained over the whole training is the same for each participant, only the difficulty level varies. Exercises were chosen to train a wide range of motor functions. Aerobic fitness was not trained to avoid confounds by induced metabolic changes [82, 83].

##### Procedure

Exercises are explained by the trainers. Each of the 72 training blocks is assigned to either the floor program or the walking program in a predetermined order until the last training session is finished: (1) walking, (2) floor, (3) walking, (4) floor, (5) floor. A floor program block contains two different strength exercises of 4 min and one balance exercise of 4 min in between. Each of the three exercises is followed by a recovery period of 1 min in which a flexibility exercise is performed. In one walking program block, three walking exercises are performed for 5 min each. Over the whole training, the different exercises are provided several times in a predefined order which is similar for all participants. The first time an exercise is presented, it is performed at the lowest difficulty level and adapted over the course of the training based on trainer’s valuation (except of the flexibility exercises which are used for physical recovery and don’t change in their difficulty level). Criteria for valuation are, e.g., unsteady stand, uncoordinated movements, tremor, heavy breathing.

#### Simultaneous cognitive-motor training

This training is conducted in the same customized exercise room as the motor training. Cognitive exercises are presented on a 48″ screen, with one separate screen per participant. Screens are mounted at eye level in front of the participants, to ensure that the visual angle of the presented exercises remains comparable to the cognitive training. Participants follow the same procedures as in the motor and cognitive training programs, but they perform the exercises simultaneously. They perform, for example, knee lifts concurrently with the N-back exercise. Both motor and cognitive exercises are adapted to the training progress as described above.

### Overall study procedure

**Fig. 3:**
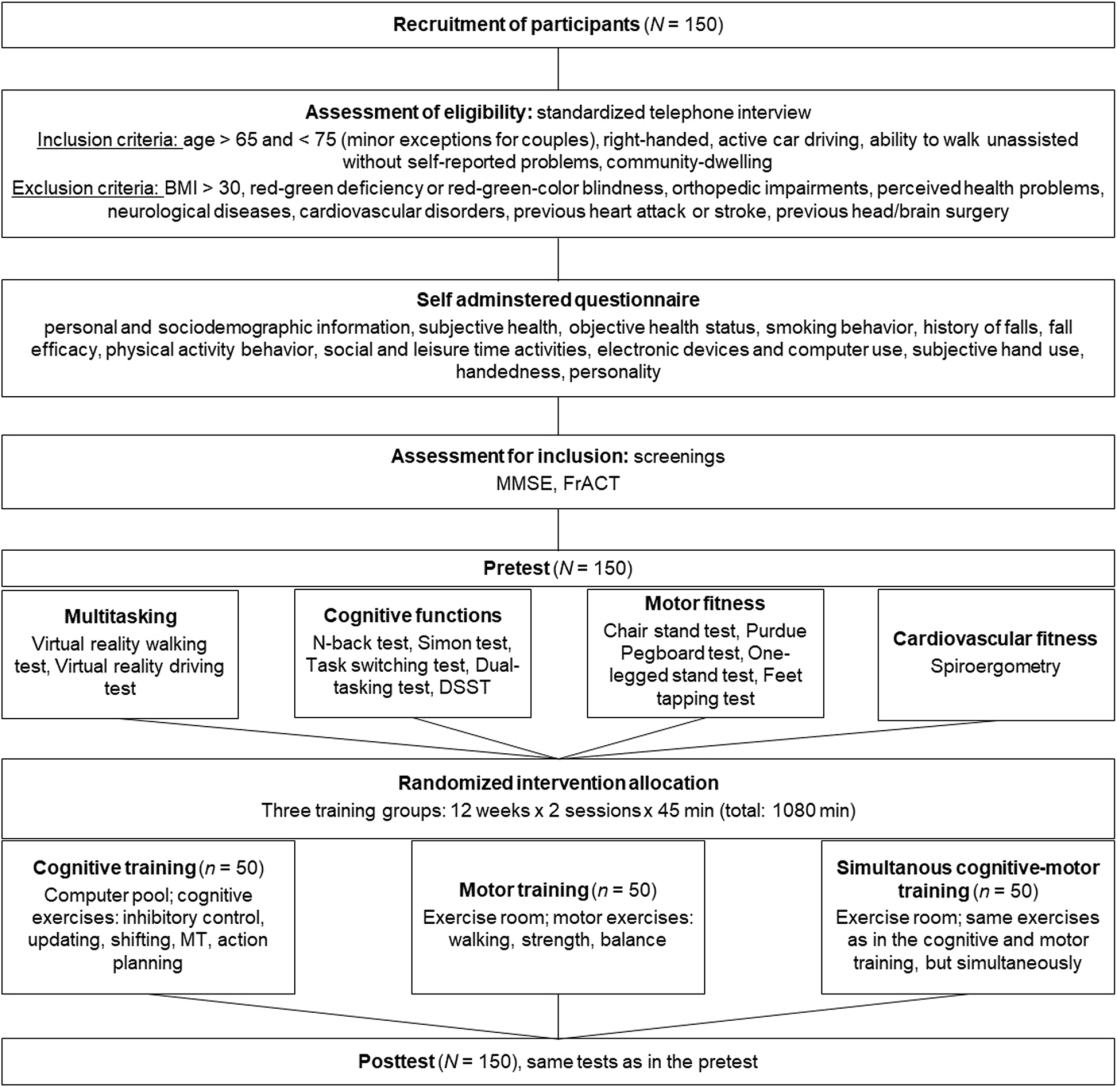
Schedule of activities. *BMI* = Body Mass Index; *DSST* = Digit Symbol Substitution Test; *FrACT* = Freiburg Visual Acuity Test; *MMSE* = Mini Mental State Examination

The overall schedule of activities is presented in Fig. 3. Pretests as well as posttests are administered on two separate days, with one rest day in between. Each testing day lasts about 2 to 2.5 hrs including small breaks and instructions. Different predetermined testing sequences are implemented to control for order effects and other potential confounds: At the beginning of each testing day, cognitive and motor tests and the visual acuity test are administered in four different orders. After that, participants are tested in the driving simulator, with driving courses presented in a counterbalanced order (cf. section on virtual reality driving test), independently of the tests administered before. The virtual reality driving test is followed either by the virtual reality walking test or by cardiorespiratory fitness assessment, also independently of the tests administered before. After their second day of pretesting, participants are informed about their training program. Training starts 1 week or less after pre-tests and is conducted for 12 weeks (two times per week, cf. section on training). Posttests are scheduled in the week after the last training session. For each given participant, posttests are scheduled in the same order as pretests.

### Data analysis plan

#### Data management

Electronic data will be stored on password protected hard drives. Hard drives and hard copy forms will be stored in locked cabinets to which only the current project staff has access. Databases will only include pseudonymized ID codes. The key-list that allows to link participant’s names with the ID codes will be stored separately from the data. Data quality will be promoted by double data entry and plausibility and range checks.

#### Statistical analysis

Our main statistical analyses evaluate whether the three training programs have differential effects on MT performance, in dependence on participants’ initial cognitive functioning and motor fitness. We calculate linear mixed-effect models. Dependent variables are MT performance in the walking and driving test. The independent variables Training Group (cognitive, motor, simultaneous cognitive-motor) and Time (pre, post), Initial Cognitive Functioning and Initial Motor Fitness (continuous variables) as well as potential confounds such as age, gender or education are added as fixed effects.

We expect significant three-way interactions between Training Group, Time and Initial Cognitive Functioning (H1), such that the interaction effect of Initial Cognitive Functioning and Time on MT performance is larger (i.e., more negative slope) in the cognitive training group than in the motor and simultaneous cognitive-motor training group. Similarly, we assume a significant three-way interaction between Training Group, Time and Initial Motor Fitness (H2), such that the interaction effect of Initial Motor Fitness and Time on MT performance is larger (i.e., more negative slope) in the motor training group compared to the cognitive or cognitive-motor training group. Lastly, we further expect a significant four-way interaction between Training Group, Time, Initial Cognitive Functioning and Initial Motor Fitness (H3). Here we assume that the triple interaction of Initial Cognitive Functioning, Initial Motor Fitness and Time on MT performance is larger (i.e., more positive slope) in the cognitive-motor training group than in the cognitive or motor training group.

All statistical analyses are performed using SPSS for Windows (IBM Corp., Armonk, NY) and R [84].

#### Sample size estimate / power calculations

To approximate the required sample size to test our hypotheses, a statistical power analysis was performed a priori with G*Power 3.0. The following parameters were entered: *f*^*2*^ = .085 (i.e., a small to medium-sized effect, [40, 85]), alpha = .05, 1-beta = .80, number of tested predictors = 2 (for each hypothesis, based on dummy coded variables). The estimated required sample size was *N* = 118 to provide a sufficient power to detect a small to moderate effect. To account for a typical attrition rate of 20% for comparable training studies [20–26, 86], we plan to recruit a total of *N* = 150 participants with training group sizes of *n* = 50 participants each.

#### Data monitoring

No data monitoring committee is required because the training is conducted by skilled and trained instructors who have no interest in a specific outcome of the trainings. Furthermore, participants are under observation of qualified project staff that intervenes if they notice symptoms such as dizziness, confusion or pain during the measurements at pre- and posttest or training.

## Discussion

Our study evaluates whether three different training programs (cognitive, motor, and simultaneous cognitive-motor training) have differential effects on MT performance, in dependence on participants’ initial cognitive functioning and motor fitness. In particular, we expect that for participants with lower cognitive functioning, MT performance benefits more from cognitive training than from motor or simultaneous cognitive-motor training, for participants with lower motor fitness, MT performance benefits more from motor training than from cognitive or simultaneous cognitive-motor training, and for participants with higher motor fitness and higher cognitive functioning, MT performance benefits more from simultaneous cognitive-motor training than from motor or cognitive training.

Our findings will be relevant, both for basic and for applied science. On the basic side, they will contribute to the long-standing debate whether MT is an emergent property, arising from the interaction of the constituent tasks (e.g., [87]), or rather is a dedicated sensorimotor processing stage [7]. Specifically, if training of the constituent tasks is indeed beneficial for MT performance, as per our hypotheses H1 and H2, this would support the emergent-property model. If such a benefit cannot be substantiated, this would rather support the dedicated-stage model.

On the applied side, our findings will provide experimental evidence for or against the notion that tailored training is more efficient than off-the-rack standardized training [41–45]. If the data are in agreement with our hypotheses, they would support this notion; otherwise, they would oppose it. More specifically, the outcome of the present study can be used to assign future participants to a training program that matches best their needs. In particular, we could use each participant’s pretest scores of the initial cognitive and motor performance to calculate the expected MT performance after cognitive training, after motor training and after cognitive-motor training. Based on these predictions, each person could then be referred to the training program that is likely to be most effective for that particular person.

## Data Availability

The anonymized datasets used and/or analyzed during the current study are available from the corresponding author on reasonable request.

## Abbreviations

BMI: Body Mass Index
DSST: Digit-Symbol-Substitution Test
DT: Dual-tasking /Dual-task
ECG: Exercise Electrocardiogram
FrACT: Freiburg Visual Acuity Test
GRAIL: Gait Real-time Analysis Interactive Lab
ISI: Inter Stimulus Interval
MMSE: Mini-Mental State Examination
MT: Multitasking / Multitask
MTC: Multitasking Costs
RER: Respiratory Exchange Ratio
RPE: Rate of Perceived Exertion
ST: Single-Tasking / Single-Task
TUC: Chemnitz University of Technology
VA: Visual Acuity
VCO2: Volume of Carbon Dioxide
VO2: Volume of Oxygen
WWU: University of Münster
